# A Nuclear mtDNA Concatemer (Mega-NUMT) Could Mimic Paternal Inheritance of Mitochondrial Genome

**DOI:** 10.3389/fgene.2019.00518

**Published:** 2019-06-06

**Authors:** Jorune Balciuniene, Darius Balciunas

**Affiliations:** ^1^Division of Genomic Diagnostics, Department of Pathology and Laboratory Medicine, The Children's Hospital of Philadelphia, Philadelphia, PA, United States; ^2^Department of Biology, Temple University, Philadelphia, PA, United States

**Keywords:** human genetics and genomics, mitochondrial DNA, paternal inheritance, biparental inheritance, mitochondrial disease

In most animal species including humans, mitochondrial genome (mtDNA) is inherited strictly uniparentally from the mother (Ladoukakis and Zouros, 2017). Specific molecular mechanisms have been found to ensure elimination of paternal (sperm) mitochondria from early embryos in species as diverse as the nematode, the fruit fly and the mouse (Rojansky et al., 2016; Sato and Sato, 2017). The precise mechanism responsible for elimination of paternal mitochondria in humans remains to be elucidated. Nonetheless, paternal contribution of mtDNA to the fertilized egg would be negligible, as mtDNA molecules in the oocyte outnumber those in a sperm by over four orders of magnitude (Pyle et al., 2015). Earlier reports claiming paternal mtDNA contribution in humans via identification of paternal haplotype in an individual or via inference of mtDNA recombination signatures from population data are scarce and remain uncorroborated independently (Awadalla et al., 1999; Kivisild et al., 2000; Schwartz and Vissing, 2002; Taylor et al., 2003). Even extreme-depth sequencing of human trios failed to provide evidence of paternal mtDNA leakage (Pyle et al., 2015).

In a recent issue of PNAS, Luo et al. (2018) presented evidence for biparental inheritance of mtDNA in three unrelated human pedigrees. High-depth sequencing of whole mtDNA from blood was performed on 17 family members spanning three generations. Thirteen individuals displayed a high level of mtDNA heteroplasmy (ranging from 24 to 76%), indicative of the presence of two mtDNA haplotypes. To exclude the possibility of sample mix-up and/or contamination, mtDNA was re-sequenced by two different CLIA-accredited laboratories on newly obtained blood samples.

Paternal inheritance of mtDNA was observed in 4 out of 6 tested offspring of three heteroplasmic fathers (one in each pedigree). The offspring were heteroplasmic for one of the paternal haplotypes in addition to the maternally inherited mtDNA haplotype: 1 out of 2 children of II:4 in Family A, 1 out of 2 children of II:3 in Family B, and both children of II:3 in Family C. The haplotype transmitted by the heteroplasmic fathers was deduced to have come from the deceased paternal ancestor of generation I of each family. Heteroplasmic mothers were expected, by default, to transmit both haplotypes to their children, which was confirmed by sequencing children of two of the heteroplasmic mothers. All 3 children of mother III:6 in Family A were heteroplasmic, while only 1 out of 2 children of mother III:6 in Family C was tested and found to be heteroplasmic (Luo et al., 2018).

Repeated paternal transmission of mitochondria at a high level of heteroplasmy in the offspring cannot be explained by known genetic mechanisms. Luo et al. suggest the presence of an autosomal dominant mutation in a gene responsible for the elimination of paternal mitochondria. They further speculate that the same mutation would not only compromise the elimination of paternal mitochondria but also lead to selective replication of a specific paternally transmitted mtDNA haplotype across several generations.

Subsequent commentary and letter pointed out that Luo et al. have not entirely ruled out the possibility of nuclear origin of sequenced mtDNA, but did not provide a clear and testable alternative hypothesis to explain the data (Lutz-Bonengel and Parson, 2019; McWilliams and Suomalainen, 2019; Vissing, 2019). Hereby we postulate that observations of Luo et al. are consistent with presence of a multicopy mtDNA concatemer that is integrated into the nuclear genome (“Mega-NUMT”) and segregates in an autosomal dominant manner regardless of its parental origin, not depending on the transmission and selective amplification of paternal mitochondria.

Our hypothesis is plausible because:

1. *In each family, it is always the same mtDNA haplotype that segregates across generations and contributes to the observed mtDNA heteroplasmy*. A nuclear mtDNA concatemer would be expected to segregate in an apparently autosomal dominant fashion, an inheritance pattern that is compatible with the family data presented in the study.
2. *Sequencing methods used in the study do not differentiate between nuclear and mitochondrial origin of DNA templates*. The DNA samples analyzed by the authors contained both mitochondrial and nuclear genomes. The long-range PCR amplification employed by the authors would successfully amplify from both genomes, resulting in sequence reads from both the mtDNA molecules as well as the postulated full length mtDNA concatemer (Figure 1).
3. *Observed level of heteroplasmy is compatible with a multicopy mtDNA concatemer (Mega-NUMT)*. Considering 100 mtDNA copies per blood cell [average number of mtDNA in blood among control individuals ranged from 50 to 136 in different reports (Mengel-From et al., 2014; Memon et al., 2017; Svendsen et al., 2019)], 75% of apparent mtDNA heteroplasmy could be imitated by a concatemer of 300 mtDNA copies. Therefore, concatemers of 100–300 copies of mtDNA (or 1.5–6 Mb in size) would be sufficient to explain the levels of heteroplasmy observed by Luo et al. (2018).
4. *Nuclear transfer of mitochondrial DNA is a well-established phenomenon in eukaryotes and is an ongoing evolutionary process*. Human genome contains >700 fixed NUMTs (nuclear sequences of mitochondrial origin) with new insertions contributing to human variation (Hazkani-Covo et al., 2010; Calabrese et al., 2012; Dayama et al., 2014).

**Figure 1:**
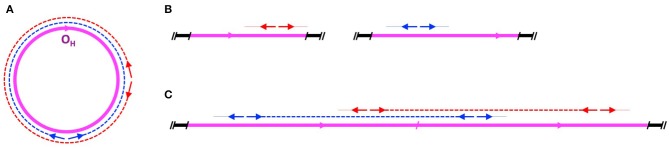
Amplification of mtDNA by long range PCR. **(A)** Amplification on a circular mitochondrial chromosome. mtDNA is shown in magenta, with the H-strand origin (O_H_) indicated by an arrowhead. Two long-range PCR primer pairs are shown as red and blue arrows, with corresponding amplicons in dashed red and blue lines. **(B)** No double-stranded amplification product would be generated from partial or single-copy NUMTs dispersed in the nuclear genome. Autosomal DNA is shown in black. Dotted lines indicate failure to amplify. **(C)** Amplification of full-length mtDNA sequences on a linear two-copy head-to-tail concatemer integrated into an autosome. Note that the amplicons in **(A)** and **(C)** are identical, making it impossible to differentiate between the long range PCR products obtained on a circular mitochondrial chromosome and a nuclear mtDNA concatemer.

Several additional indirect lines of evidence support our hypothesis. Instances of multimeric NUMTs have been reported previously: a partial 7.9-kb mtDNA segment of 38–76 tandem copies is present in the nuclear genomes of domestic cats (Lopez et al., 1994), while complex NUMTs patterns are common in plants (Hazkani-Covo et al., 2010). The latter is believed to be the result of the concatemerization of organelle (mitochondrial and plastid) DNA prior to nuclear integration (Richly and Leister, 2004). Moreover, catenated mtDNA structures have been reported in normal human cardiac tissue (Pohjoismäki et al., 2009), and mtDNA concatemers that were presumed to be a product of rolling circle amplification have been observed in human fibroblasts exposed to reactive oxygen species (Ling et al., 2016). These observations suggest that mtDNA concatamers do occur, and their integration into the nuclear genome is possible. While a concatemer of 100+ copies could be considered unusually large, concatemers of such size range have been observed in animal transgenesis experiments (Garrick et al., 1998; McGrail et al., 2011; Yong et al., 2015).

Our hypothesis can be readily tested using methods that differentiate between mtDNA sequences of nuclear and mitochondrial origin. One way would be to separate cytoplasmic (mitochondria-containing) and nuclear fractions prior to DNA extraction, PCR amplification and sequencing. Also, we would expect that due to differences between nuclear and mitochondrial transcription machinery the “nuclear” mtDNA haplotype would be underrepresented in total cellular RNA, as detectable by RT-PCR or RNA-Seq. Finally, a multicopy concatemer may be detectable by fluorescence *in situ* hybridization (FISH) as previously reported for identification of concatemeric transgenes in mouse cells (Schubert and Schmidtke, 2010).

While the scenario we propose is hypothetical, it is not as paradigm-changing as the high-level, selective inheritance of paternal mitochondria would be. It should therefore be formally ruled out before asserting the biological and evolutionary impact of paternal inheritance of mitochondrial genome.

## Author Contributions

All authors listed have made a substantial, direct and intellectual contribution to the work, and approved it for publication.

### Conflict of Interest Statement

The authors declare that the research was conducted in the absence of any commercial or financial relationships that could be construed as a potential conflict of interest.
